# When nematodes take flight: Multi-scale dispersal of the beech leaf disease nematode in forest ecosystems

**DOI:** 10.1371/journal.pone.0361390

**Published:** 2026-10-26

**Authors:** Erika Consoli, Lily Berry, Niclas Lyndby, Miriam R. Wayman, Lucas W. DeGroote, Andrea M. Lindsay, Danielle Martin, Camelia Kantor, Mihail Kantor

**Affiliations:** 1 Department of Plant Pathology and Environmental Microbiology, The Pennsylvania State University, University Park, Pennsylvania, United States of America; 2 Powdermill Nature Reserve, Rector, Pennsylvania, United States of America; 3 United States Department of Agriculture, Forest Service, Forest Health Protection, Morgantown, West Virginia, United States of America; 4 Huck Institutes of the Life Sciences, The Pennsylvania State University, University Park, Pennsylvania, United States of America; University of Limpopo, SOUTH AFRICA

## Abstract

Beech leaf disease, caused by the invasive foliar nematode *Litylenchus crenatae*, is an emerging threat to *Fagus grandifolia* across North America. Since its initial detection in 2012, the disease has spread to a total of 16 U.S. states and the Canadian Province of Ontario; however, the mechanisms underlying nematode dispersal remain poorly understood. This study evaluated multiple potential dispersal pathways across two forest sites in central Pennsylvania through integrated field surveys, environmental monitoring, and experimental validation. Weekly sampling was conducted to characterize nematode population dynamics in relation to temperature, precipitation, wind, and humidity. Long distance water-mediated dispersal was assessed through creek sampling, while biotic dispersal was investigated through field sampling from migratory bird feet during high-moisture events and a controlled greenhouse transmission experiment. Wind dispersal was further examined in a pilot study using unmanned aerial vehicle-assisted rainwater collection during precipitation events. Nematode presence was detected across all investigated pathways, supporting dispersal via water flow, rain splash, and biotic vectors. Funnel-based sampling recovered *Litylenchus crenatae* up to 90 m from infected trees, demonstrating movement beyond the immediate canopy area. Confirmed downstream transport of nematodes suggests waterways may facilitate long-distance dispersal across forested landscapes. *Litylenchus crenatae* was recovered from the feet of migratory birds, and greenhouse experiments confirmed that bird-associated nematodes could infect healthy beech seedlings, providing evidence for avian-mediated transmission. Desiccation assays demonstrated that live *Litylenchus crenatae* specimens could survive up to 10 days in the absence of available moisture, indicating that the nematodes may remain viable during transport on bird feet or exposed surfaces. In addition, unmanned aerial vehicle-assisted rainwater sampling detected *Litylenchus crenatae* within canopy-associated precipitation samples, providing additional evidence for short-distance aerial dispersal during wind and rain events. Together, these findings demonstrate that beech leaf disease spread is driven by multiple interacting abiotic and biotic dispersal mechanisms operating across spatial scales.

## Introduction

Beech leaf disease (BLD) has emerged as a major threat to temperate forest ecosystems in North America, resulting in decline and mortality of American beech (*Fagus grandifolia*) populations. First detected in the United States near Lake Erie, Ohio, in 2012 [1], BLD has expanded rapidly across eastern North America, with detections extending across a broad geographic range within little more than a decade. The expansion has been strongly asymmetric, with the known distribution extending approximately 1,200 km northeast and 250 km southeast from the initial detection area. This pronounced multidirectional spread highlights the capacity of BLD to move rapidly across the landscape and underscores the threat of continual expansion into currently unaffected beech forests [2,3]. Infected trees exhibit characteristic foliar banding, reduced photosynthetic capacity, canopy thinning, and, in severe cases, mortality [4]. Given the ecological importance of American beech in maintaining forest structure, producing mast that supports wildlife communities, and contributing to nutrient and carbon cycling in eastern North American forests [4]; widespread decline caused by beech leaf disease could have significant ecosystem-level consequences [2,5,6].

BLD is caused by the nematode *Litylenchus crenatae* (*L. crenatae*), an invasive plant-parasitic species first reported in association with beech trees in Japan [7]. *L. crenatae* belongs to the family Anguinidae, a group of nematodes known to parasitize aboveground plant tissues [8]. Similar to other groups of plant-parasitic nematodes, *L. crenatae* can go through anhydrobiosis, a survival strategy that enables them to withstand desiccation and other environmental extremes [9]. Although the disease cycle is not yet fully understood, *L. crenatae* is known to overwinter in beech buds, where feeding activity induces cellular enlargement in bud scales [4,10]. This feeding disrupts plant cell wall integrity, leading to physiological and developmental stress changes including chlorophyll reduction; resulting in the characteristic banding and crinkling of leaves upon leaf emergence in spring [10]. Nematode populations increase during the spring growing season and typically decline during summer, with resurgence occurring in early fall [11].

While the pathogenic role of this nematode is well established [4], the mechanisms underlying its dispersal within and among forest stands remain poorly understood. This knowledge gap limits our ability to predict disease spread and develop effective management strategies. Previous studies [11,12] demonstrated temporal variation in nematode populations within infected beech trees, with the latter suggesting that environmental factors, such as moisture and temperature, influence nematode activity and detection. However, these studies have primarily focused on localized population dynamics and have not fully addressed how nematodes disperse across broader spatial scales, particularly over long distances or among disconnected forest patches and isolated beech trees.

Several dispersal pathways have been hypothesized for *L. crenatae* (see graphical abstract). Abiotic mechanisms, including rain splashes, wind, and water flow, may facilitate short- and long-distance movement of nematodes [12]. Rain events, in particular, could enable dispersal from infected canopies to surrounding vegetation, while water streams such as creeks may act as corridors for downstream transport. At the same time, biotic vectors such as birds and other animals may contribute to dispersal by internally and externally carrying nematodes, especially under high-moisture conditions. Although these mechanisms are plausible, empirical evidence supporting their relative importance remains limited.

Understanding dispersal in this system requires an integrated framework that considers both abiotic and biotic pathways operating across multiple spatial and temporal scales. Forest pathogens often rely on a combination of mechanisms rather than a single dominant vector, and interactions among environmental conditions, host distribution, and vector activity can strongly influence disease dynamics. For BLD, identifying the relative contribution of these pathways is critical for explaining its rapid spread, anticipating future expansion under changing temperature conditions, and helping ensure the survival of beech forests.

In this study, we investigated multiple potential dispersal pathways of *L. crenatae* across two forest sites during 2024 and 2025, representing different spatial and temporal scales of beech leaf disease spread. Building on previous monitoring efforts by [12], we combined weekly nematode sampling with environmental data collection to characterize spatiotemporal population dynamics and identify environmental factors associated with nematode movement and dispersal. In addition, a novel pilot study was conducted to evaluate the potential for aerial dispersal of *L. crenatae* through wind- and rain-mediated movement. To investigate whether nematodes could be transported at different vertical strata within and above the forest canopy, collection assisted by an unmanned aerial vehicle (UAV) was performed during precipitation events. Rainwater samples were collected at multiple heights beneath and above the tree canopy to assess the presence of *L. crenatae* associated with airborne water movement under natural environmental conditions.

Waterways such as creeks and streams may also represent important long-distance dispersal pathways for *L. crenatae* within forest ecosystems. To evaluate water-mediated long-distance dispersal, water stream sampling was conducted at increasing distances downstream from infected beech trees. The assumption was that infected beech buds, leaves, and subsequent rain runoff may enter flowing water and be transported downstream, potentially introducing inoculum into previously uninfected areas. In addition, stream-associated nematodes may provide a secondary source of inoculum for transport by birds and other wildlife interacting with aquatic environments.

The potential role of birds as dispersal agents of *L. crenatae* was assessed under field conditions through the collection and analysis of bird foot wash samples during fall migratory periods. To further validate the capacity for bird-mediated transmission, controlled greenhouse experiments were conducted to simulate nematode transfer and subsequent host infection. Because successful *L. crenatae* dispersal on bird feet may depend on the nematode’s ability to tolerate temporary moisture loss, laboratory desiccation assays were also performed to evaluate the long-term survival of *L. crenatae* under dry conditions. These experiments provided insight into how long nematodes may remain viable in the absence of moisture and, consequently, their potential to survive transport on bird feet under natural conditions.

Together, by integrating both observational and experimental approaches, this study provides a comprehensive overview of the mechanisms potentially contributing to the local and long-distance spread of BLD in forest ecosystems. Specifically, this study aimed to (i) quantify the spatiotemporal patterns of nematode dispersal in relation to environmental variables, (ii) evaluate the potential for water-mediated dispersal, (iii) determine whether birds can act as dispersal vectors, and (iv) assess *L. crenatae* tolerance to desiccation as a mechanism that may enhance survival during dispersal.

## Materials and methods

### *Litylenchus crenatae* short distance dispersal

#### Research sites.

This study was conducted at two locations within Stone Valley Forest, Pennsylvania, under a research agreement with the Pennsylvania State University Forestlands Management Office. Both sites consisted of forest stands containing BLD-symptomatic American beech trees located 1.15 km apart. The first location, hereafter referred to as Site 1 (40°37′51.3″ N, 77°53′08.03″ W), is situated near a water stream (creek) and represents a continuation and expansion of the study [12]. The second location, hereafter referred to as Site 2 (40°37′28.34″ N, 77°54′12.8″ W), is situated in an area without flowing water and away from nearby roads. Although both sites contained infected trees, each was used to evaluate the dispersal of *L. crenatae* at varying distances from infected trees.

#### Experimental design and environmental data.

The experimental design at Site 1 followed the approach previously described [12], with sampling extended through the 2024 and 2025 growing seasons. To investigate dispersal over greater spatial scales, Site 2 was established using a distance-gradient design centered around a single infected beech tree.

In summary, at Site 1, four wooden stands were installed, each supporting three funnels positioned 40 cm above the ground level. Stands were placed as described [12]. At Site 2, a linear series of six wooden stands at a height of 40 cm from the ground, each containing three funnels with a diameter of 13 cm, were established at increasing distances from a single infected tree (about 50% of canopy infected and a Diameter at Breast Height – DBH: 1.6 m). The stands were placed at 0 m, 30 m, 60 m, 90 m, 120 m, and 150 m from the infected tree, and this configuration was designed to quantify the horizontal dispersal potential of *L. crenatae* associated with beech trees.

Each funnel consisted of a modified Baermann apparatus fitted with rubber tubing and a clamp. Funnels were filled with tap water and covered with metal mesh to prevent large debris from entering. At Site 1, samples were collected weekly from May 8 to November 6, 2024, and from May 9 to November 21, 2025. At Site 2, samples were collected every three days from August 18 to November 19, 2024, and from August 22 to November 17, 2025. Sampling was performed by opening the bottom clamp and collecting the funnel contents into 50 mL centrifuge tubes. Then, the clamp was closed, and fresh tap water was added to each funnel.

Local weather data was obtained from a nearby weather station located at Shaver’s Creek Environmental Center in Huntingdon County, approximately 4.5 km from both Site 1 and Site 2. The station recorded hourly environmental measurements throughout the 2024 and 2025 sampling periods. Environmental variables, including total and average precipitation, wind speed and direction, relative humidity (RH), dew point, leaf wetness index (LWI), and temperature were evaluated as potential factors influencing nematode dispersal from infected beech trees.

#### Assessment of *L. crenatae* aerial dispersal using unmanned aerial vehicles.

A remotely operated unmanned aerial vehicle (UAV; Fisherman FD1 + , Swellpro, China) was used to evaluate the potential dispersal of *L. crenatae* in the immediate airspace around infected beech canopies (Fig 1). This study was conducted at the Penn State Research Forest, Blue Lick Hollow Rd (40°37′57.012″ N, 77°53′16.441″ W). The site was selected due to the accessibility provided by the adjacent roadway and the presence of an isolated infected beech tree near the flight area. Due to the relatively few precipitation events that occurred during the fall 2025 sampling period, this study was limited to a small number of collection events and was intended primarily to validate the potential for wind- and rain-mediated aerial movement of *L. crenatae* under natural field conditions.

**Fig 1:**
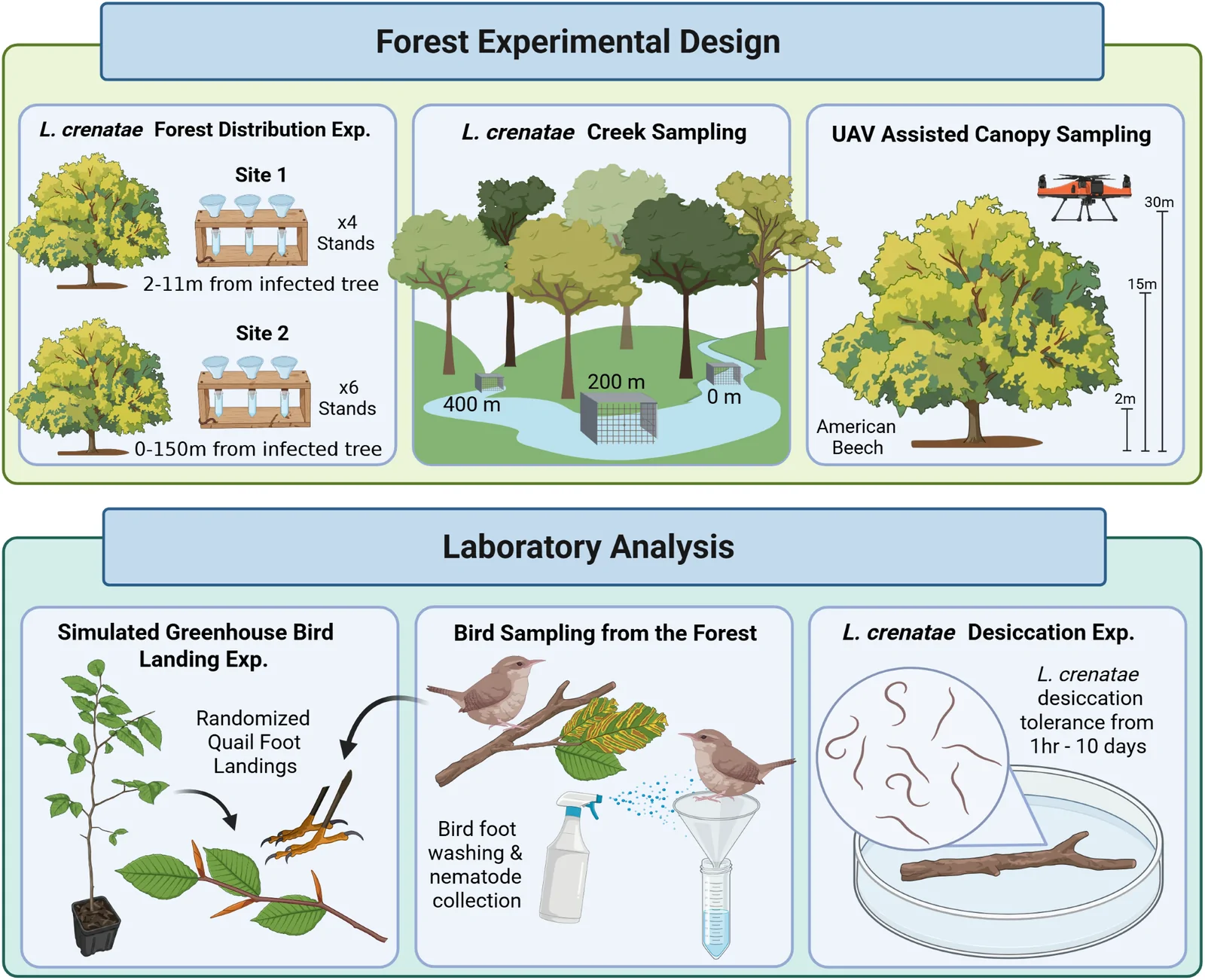
Experimental design used to investigate environmental dispersal of *Litylenchus crenatae.* Field experiments included (top left) monitoring *L. crenatae* dispersal using funnel traps positioned at increasing distances from *L. crenatae* infected American beech (*Fagus grandifolia*) trees at two forest locations (Site 1 and Site 2) (Top middle) sampling stream water at three locations along a forest creek; and (Top right) Unmanned aerial vehicle (UAV)-assisted canopy sampling during precipitation events at heights of 2, 15, and 30 m. Laboratory experiments included (bottom left) a simulated bird-landing assay using quail feet to evaluate nematode transfer to beech foliage; (bottom middle) collection of nematodes from the feet of wild migratory birds captured in the forest; and (bottom right) desiccation assays to assess *L. crenatae* survival on beech twigs under dry conditions by recording active and inactive nematodes.

The UAV (S1 Fig) was outfitted with custom-built plexiglass collection chambers (S1A Fig; dimensions 5 cm H: 8.5 cm W: 25.5 cm L) mounted on the sides of the aircraft to capture nematodes, insects, and other airborne debris encountered during flight. Before each flight, the inner surfaces of the collection chambers were lightly sprayed with tap water to facilitate adhesion of airborne nematodes. Flight collections were conducted during precipitation and high-moisture events on October 8, October 30, and November 14, 2025. A total of 17 flight collection events were conducted across these three dates, with five collections on October 8, nine on October 30, and three on November 14. The number of collections varied among dates because consistent rainfall was not available throughout the sampling period.

The UAV was operated beneath and adjacent to the canopy of an infected beech tree (1.2 m DBH; approximately 20 m height) at vertical flight ranges of 2 m, 15 m, and 30 m, while maintaining an approximate horizontal distance of 12 m from the tree trunk. At each height, the UAV hovered in place for 2 min while executing a continuous yaw rotation to maximize airborne particle collection. Following each flight, the collection chambers were detached from the UAV, and their contents were rinsed into 50 mL centrifuge tubes for subsequent nematode quantification and morphological and molecular confirmation.

### *Litylenchus crenatae* long-distance dispersal

#### Assessment of *L. crenatae* dispersal via water streams.

To evaluate the potential for water stream-mediated dispersal of *L. crenatae*, nematode collection traps were installed along a creek (Fig 1) running through Site 1 described above. This configuration allowed the assessment of *L. crenatae* movement originating primarily from the infected stand through leaf fall and rain-driven runoff entering the creek system. Field observations further confirmed the presence of infected beech leaves within the creek below the infected stand.

Three collection traps were constructed using stacked 17.8 cm diameter mesh sieves (#230, #60, and #10; Eisco Labs, EIS-BI0139S4), arranged with the finest mesh positioned at the bottom of the stack. The traps were positioned in the creek in an area with consistent water flow with the sieve opening facing upstream. The first trap was installed directly beneath the infected beech stand, while subsequent traps were positioned 200 m and 400 m downstream to assess the extent of nematode movement along the creek system.

Due to natural variation in stream depth and water discharge throughout the sampling period, the traps intercepted only a small portion (approximately 1/5) of the total creek flow, with water levels often insufficient to fully submerge the sieve assembly (S2 Fig). Consequently, the system functioned as a passive subsampling approach intended to capture representative nematode movement within the stream, rather than the total water volume passing through the creek.

Samples were collected weekly from September 5 to November 19, 2025. During each sampling event, the bottom sieve was carefully rinsed with tap water to recover accumulated sediment and nematodes. Sediments and nematodes were collected into 50 mL centrifuge tubes and transported to the laboratory for processing. Nematodes were recovered from the collected material using the sugar flotation method [13], then identified and quantified under an inverted microscope (Axiovert 5, Zeiss, Jena, Germany) at 40 × magnification. Specimens identified as *L. crenatae* were subsequently confirmed by molecular analysis.

#### Assessment of bird-mediated nematode dispersal.

To evaluate whether birds serve as vectors of the beech leaf disease nematode, we partnered with Powdermill Nature Reserve in Rector, Westmoreland County, Pennsylvania. Powdermill operates an established, long-term bird banding station that provides a unique opportunity to sample a diverse assemblage of birds under standardized and ethically approved protocols.

This research involved mist-netting and banding passerines, but no experiments on or collection of birds. Procedures were followed in accordance with state and federal legislation. This research was conducted using ethical guidelines following the North American Banding Council code of ethics (http://www.nabanding.net/banders-code-of-ethics/) and the Ornithological Council’s guidelines to the use of wild birds in research http://naturalhistory.si.edu/BIRDNET/documents/guidlines/Guidelines_August2010.pdf). We conducted mist-netting and banding under Powdermill Nature Reserve’s federal permit #08231, issued by the U.S. Geological Survey, and Pennsylvania state permit #47050, issued by the Pennsylvania Game Commission.

The mist netting area encompasses a 10-ha area dominated by old fields, marsh, and forest edge habitat, and is surrounded by forests that are becoming increasingly impacted by BLD. The banding station was operated five days per week during the fall 2025 migration season. Mist nets (n = 67) were opened 30 minutes before sunrise and checked every 40 minutes for six hours.

We coordinated our sampling efforts with routine bird banding activities, accompanying field staff during bird retrieval from the mist nets. Immediately upon removal from the nets, each bird’s feet were gently rinsed with tap water over a funnel connected to a 15 mL centrifuge tube to collect the wash for subsequent nematode analysis (Fig 1).

Sampling was conducted on three occasions during high-moisture events to maximize the likelihood of detecting externally transported nematodes. Two sampling events occurred during or immediately following rainfall (September 5 and September 23, 2025), whereas the third took place under conditions of high relative humidity (~ 98%) without precipitation (October 15, 2025). Additional sampling events could not be conducted due to a severe drought and the lack of rainfall during the fall migration banding period. Samples were labeled, linked to individual birds, and transported to the laboratory for analysis. The entire sample volume was examined for nematode presence using an inverted microscope (Axiovert 5, Zeiss, Jena, Germany) at 40 × magnification. Nematodes were identified as *L. crenatae* or other nematodes (i.e.,; any other nematode but *L. crenatae*) based on morphological characteristics, and specimens identified as *L. crenatae* were subsequently confirmed by molecular analysis as described below. Captured birds were identified to species level by bird banding staff, and both common and scientific names were recorded. Bird species associated with vectoring *L. crenatae* or other nematodes were subsequently determined.

#### Assessment of *L. crenatae* desiccation tolerance.

To evaluate the activity and potential survival capacity of *L. crenatae* under dry conditions, a laboratory desiccation experiment was conducted to simulate *L. crenatae* exposure to moisture deprivation after *L. crenatae* leaves infected beech tissues. This experiment was designed to assess how long *L. crenatae* could remain active in the absence of free moisture, a condition that may occur during transport on exposed surfaces such as bark, forest litter, soil, or animal vectors. *Litylenchus crenatae* inoculum was obtained by dissecting infected American beech buds obtained from Site 1, and desiccation time served as the experimental treatment.

Prior to selecting the American beech tree used as the source of twigs, buds and leaves from candidate trees were dissected and evaluated for the presence of *L. crenatae*. The tree selected for the experiment was located on the Penn State University, University Park campus, and neither *L. crenatae* nor other nematodes were detected in the plant material examined. For each treatment, a fresh 10-cm American beech twig was washed with tap water and allowed to air dry before use and then placed in an uncovered 60-mm Petri dish. A suspension containing 100 nematodes (all life stages) in 100 µL of water was then pipetted onto the twig and left uncovered to desiccate. Each desiccation period (1, 2, 5, 24, 48, 72, 120, and 240 h) was conducted using a separate twig and a new batch of nematodes. The inoculated twigs were maintained at room temperature for the assigned exposure period before nematode recovery and viability assessment. The experiment was conducted with three replicates per desiccation period and repeated twice.

Following each desiccation period, tap water was added to the Petri dish until the twigs were fully submerged, and samples were allowed to rehydrate at room temperature for 24 h. Then, the water was collected, and *L. crenatae* motility was quantified under an Axiovert microscope (Zeiss GmbH, Jena, Germany). Nematodes were categorized as active or inactive based on the presence or absence of movement and response to touch.

#### Greenhouse simulation of bird-mediated beech infection.

To validate field observations that birds can transport *L. crenatae* and initiate beech leaf disease infections, a greenhouse experiment was conducted simulating bird landings on healthy American beech trees. Beech saplings were maintained individually in 2.6 L plastic pots measuring 16 cm in diameter at the top, 16 cm in height, and 13 cm in diameter at the bottom. Because the use of live birds was not feasible, frozen *Coturnix* quail (*Coturnix vulgaris japonica)* feet purchased from a pet store were used instead to mimic bird contact with branches.

Infected beech leaves and buds collected from symptomatic trees at Site 1 in early fall 2025 were used to obtain *L. crenatae* inoculum for the quail feet experiment. Buds were dissected under a stereomicroscope, whereas leaves were cut into 3–5 mm strips, and *L. crenatae* nematodes were extracted using a modified Baermann pan method [14]. Extracted nematodes were pooled and transferred to a modified Baermann system to separate active from inactive individuals. Nematodes were stored for up to four days prior to inoculation at 5 °C, and the suspension concentration was adjusted to 2,000 *L. crenatae* mL^−1^. Preliminary footprint dip assays indicated that each “landing” of the quail foot deposited an average of 15 ± 6.5 *L. crenatae* individuals.

Prior to inoculation, all trees were sprayed with tap water until all leaves and branches were wet to mimic rain events. For inoculation, a detached quail foot, thawed to experimental temperature, was dipped into the *L. crenatae* stock solution and immediately contacted beech branches to simulate bird landings through two methods. In the first inoculation method, the bird foot contacted each tree at 10 randomly selected branch locations, whereas in the second method, each tree received three bird contacts near the apical buds of three randomly selected branches, resulting in an estimated inoculum level of approximately 150 ± 65 *L. crenatae* tree^-1^ and 135 ± 558 *L. crenatae* tree^-1^, respectively. Each inoculation method was applied to 15 treatment trees.

Following inoculation, trees were enclosed in 45-gallon translucent plastic bags to maintain high humidity. Bags were supported with bamboo stakes to prevent direct contact with foliage, and trees were maintained in a shaded greenhouse at 20–25°C for three days before bag removal. *L. crenatae* infection was assessed in buds at 72–74 days post-inoculation and in leaves at 180 days post-inoculation. For the bud assessment, buds were excised individually and subsequently dissected under a stereomicroscope to quantify total *L. crenatae* abundance and record *L. crenatae* motility. Leaf infection was evaluated following full leaf expansion by recording the number of infected and healthy leaves, with leaves considered infected regardless of symptom severity.

#### Molecular confirmation of *L. crenatae* nematodes.

Nematodes morphologically identified as belonging to the genus *Litylenchus* from the different studies herein were individually picked under the dissecting microscope. For molecular confirmation of morphological identification, genomic DNA was extracted from single nematode specimens using a modified Proteinase K protocol [15,16]. The D2–D3 expansion segments of the large subunit ribosomal DNA (LSU; 28S) were amplified using primers D2A (5′-ACA AGT ACC GTG AGG GAA AGT TG-3′) and D3B (5′-TCG GAA GGA ACC AGC TAC TA-3′) [16]. PCR amplification products were separated on 1% agarose gel, stained with GelRed (Biotium, CA, USA) and visualized under UV light. PCR products were purified using a QIAquick PCR Purification Kit (Qiagen, Hilden, DE), and sequenced bidirectionally using the amplification primers at the Pennsylvania State University Genomics Core Facility (University Park, PA, USA). Results were blasted against the National Center for Biotechnology Information (NCBI) GenBank database.

#### Statistical analysis.

All statistical analyses were conducted in R version 4.4.3 [17] using R studio (v.4.5.3). Environmental predictors of *L. crenatae* dispersal include precipitation, average temperature, relative humidity, leaf wetness index (LWI), wind speed, and wind direction. Wind direction was transformed into sine and cosine components to account for its circular nature. Continuous environmental variables were standardized before modeling to facilitate coefficient comparison among predictors.

Because Site 1 and Site 2 differed in spatial sampling design and distance structure, environmental models were evaluated separately for each site. Generalized linear mixed-effects models (GLMMs) with a negative binomial error distribution were fitted using the *glmmTMB* package [18] to account for overdispersion typical of count data. In all models, *L. crenatae* counts were treated as the response variable, environmental predictors were included as fixed effects, and sampling date and distance from infected trees were incorporated as random intercepts to account for repeated temporal sampling and spatial structure.

Model significance was evaluated using Type II Wald chi-square tests implemented in the *car* package [19]. Model fit and assumptions were assessed using simulation-based residual diagnostics with the *DHARMa* package [20]. Multicollinearity among environmental predictors was evaluated using variance inflation factors (VIF) implemented in the *performance* package [21]. All predictor variables exhibited low to moderate collinearity (VIF < 3.5), indicating acceptable independence among explanatory variables.

To further evaluate the relative importance of environmental variables influencing *L. crenatae* dispersal, a regression-based random forest analysis was conducted using the *random Forest* package v. 4.7-1.2 [22]. Random forest models were fitted using 500 trees (ntree = 500) and three randomly selected predictors at each node split (mtry = 3). Variable importance was assessed using mean decrease in accuracy (MDA), which quantifies the reduction in model predictive accuracy when values of a given predictor are permuted. Variables producing larger decreases in accuracy were interpreted as having greater influence on *L. crenatae* abundance and dispersal dynamics. In addition, Spearman correlation was used to correlate *L. crenatae* counts with environmental variables.

Desiccation survival data was analyzed using a generalized linear mixed-effects model (GLMM) with binomial error distribution and logit link function. The number of active and inactive *L. crenatae* recovered after rehydration was used as the response variable, with desiccation time included as a fixed effect and replicated as a random effect. Pairwise comparisons among desiccation intervals were performed using Tukey-adjusted estimated marginal means. Analyses were conducted in R studio v 4.4.3 using the packages *lme4* and *emmeans*.

Fig 1 summarizes our experimental design. This methodology presents several innovative approaches to studying the short and long-distance dispersal of beech leaf disease. Notably, it is the first study to utilize UAV for the collection of nematodes in the forest. It is also first to simulate nematode transmission via avian vectors by performing bird foot washes and controlled quail feet inoculations of greenhouse trees. Ultimately, this methodology establishes a vital link between natural and controlled systems, translating observational knowledge from forest environments into testable transmission assumptions within greenhouse, aviary, and laboratory settings.

## Results

### Short distance *L. crenatae* dispersal recovered from funnel stands

Our results from both Sites 1 and 2 confirm that *L. crenatae* dispersal occurs during two distinct seasonal periods, with the greatest migration observed during spring and fall. Sampling at Site 1, which began in early May, captured the full cycle of *L. crenatae* emergence, maturation, and dispersal. The first periods of detectable *L. crenatae* activity occurred between May 8 to June 12, 2024, and between May 9 to May 30, 2025 (Fig 2). These periods coincided with bud swelling and bud break in American beech, when expanding leaves emerge from bud scales. Spring *L. crenatae* collections ranged from as few as 5 to as many as 374 *L. crenatae* recovered within a single week. Mean weekly spring collections averaged 156 *L. crenatae* specimens in 2024, whereas substantially lower numbers were observed during spring 2025, with a weekly average of 37 *L. crenatae*. During the summer months, no *L. crenatae* were recovered from the funnel traps, indicating a temporary cessation of dispersal activity until late August.

**Fig 2:**
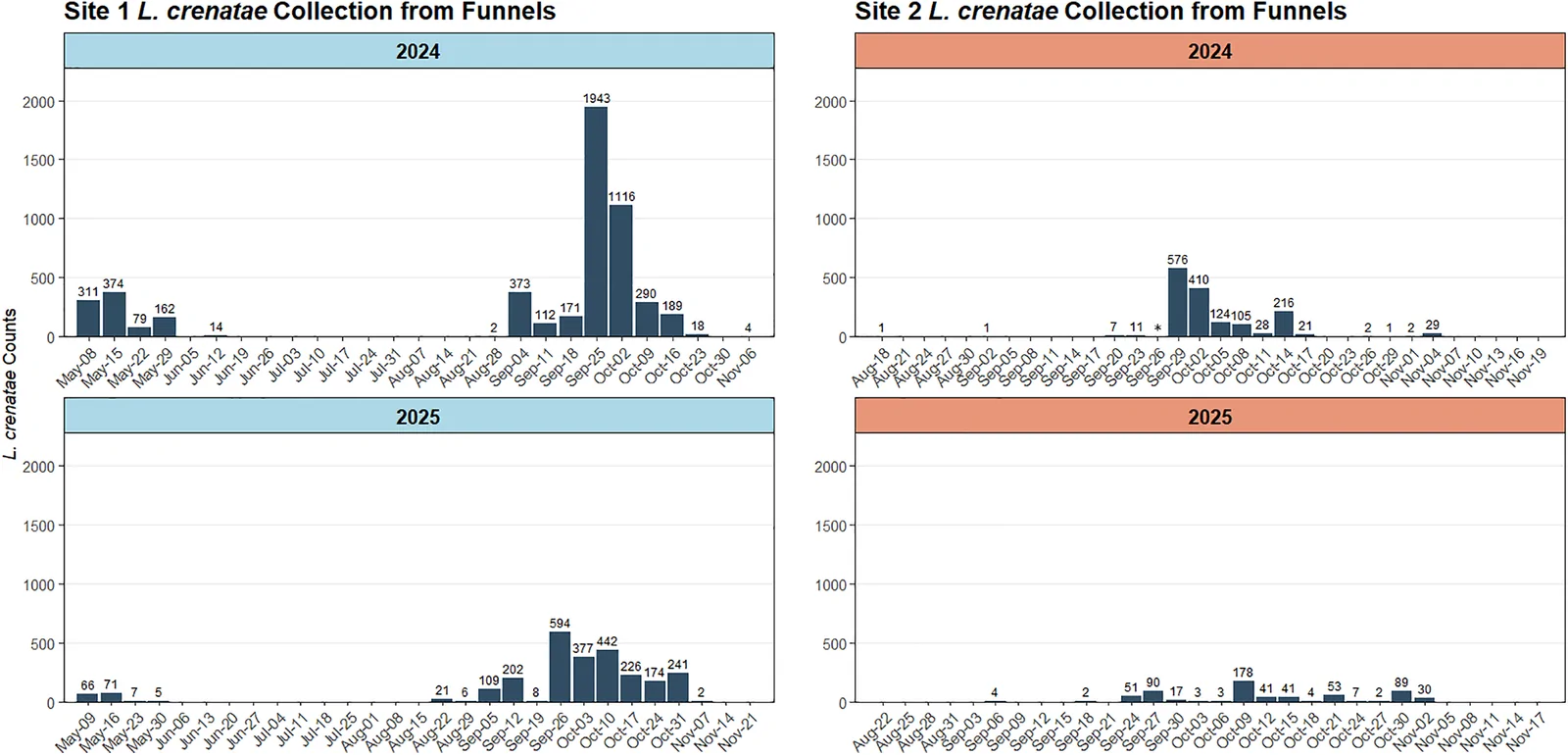
Total number of *Litylenchus crenatae* collected in funnel stands during the 2024 and 2025 seasons at (Left) Site 1 and (Right) Site 2 locations. * Note: No samples were collected at Site 2 on September 26, 2024, because the funnel traps were knocked over.

From late August through early November, *L. crenatae* movement resumed and became highly variable. Initial fall detections occurred during the final week of August, followed by a steady increase in nematode abundance throughout September, coinciding with peak leaf senescence. The highest level of activity at Site 1 was recorded during September 25, 2024, when more than 1,900 *L. crenatae* specimens were recovered in a single week. Fall 2025 collections were comparatively lower, with a maximum of 594 *L. crenatae* specimens recovered during the week of September 26, 2025. Following the September peak, *L. crenatae* dispersal gradually declined throughout the remainder of the fall season and continued tapering off until late November.

At Site 2, sampling was conducted exclusively during the fall season and was designed to evaluate dispersal over greater distances, where nematode densities would be expected to decline due to dispersal limitations and environmental exposure. Overall, fewer *L. crenatae* specimens were recovered at Site 2 compared to Site 1 in both 2024 and 2025. This reduced recovery is likely attributed to the proximity of the collection stands to an infected beech tree, as Site 2 was specifically established to evaluate longer-distance dispersal from a single infected beech tree. Despite overall lower counts, Site 2 exhibited similar seasonal trends to those observed at Site 1, with *L. crenatae* detections increasing during early fall and peaking during periods of leaf senescence, suggesting that long-distance movement may occur under favorable environmental conditions, albeit at substantially lower densities.

*Litylenchus crenatae* dispersal via environmental factors was detected at distances of up to 90 m, although most dispersal occurred within 30 m. At Site 2, funnels placed directly under the trees had the highest rate of *L. crenatae* recovery, with 903 *L. crenatae* collected throughout the entire sampling season in 2024, with a slight decrease to 606 *L. crenatae* in 2025 (Fig 3). At 30 m away from the stand, a dramatic decrease was observed in the number of *L. crenatae* collected, to only 13 and 7 *L. crenatae* nematodes recovered in the entirety of the 2024 and 2025 seasons, respectively. The farthest recorded distance that *L. crenatae* was recovered from was up to 90 m away from the infected beech stand site, with a single active *L. crenatae* nematode found on October 31^st^, 2025, at Site 2. Past 90 m, no *L. crenatae* were found in any of the funnels, although other nematodes were present.

**Fig 3:**
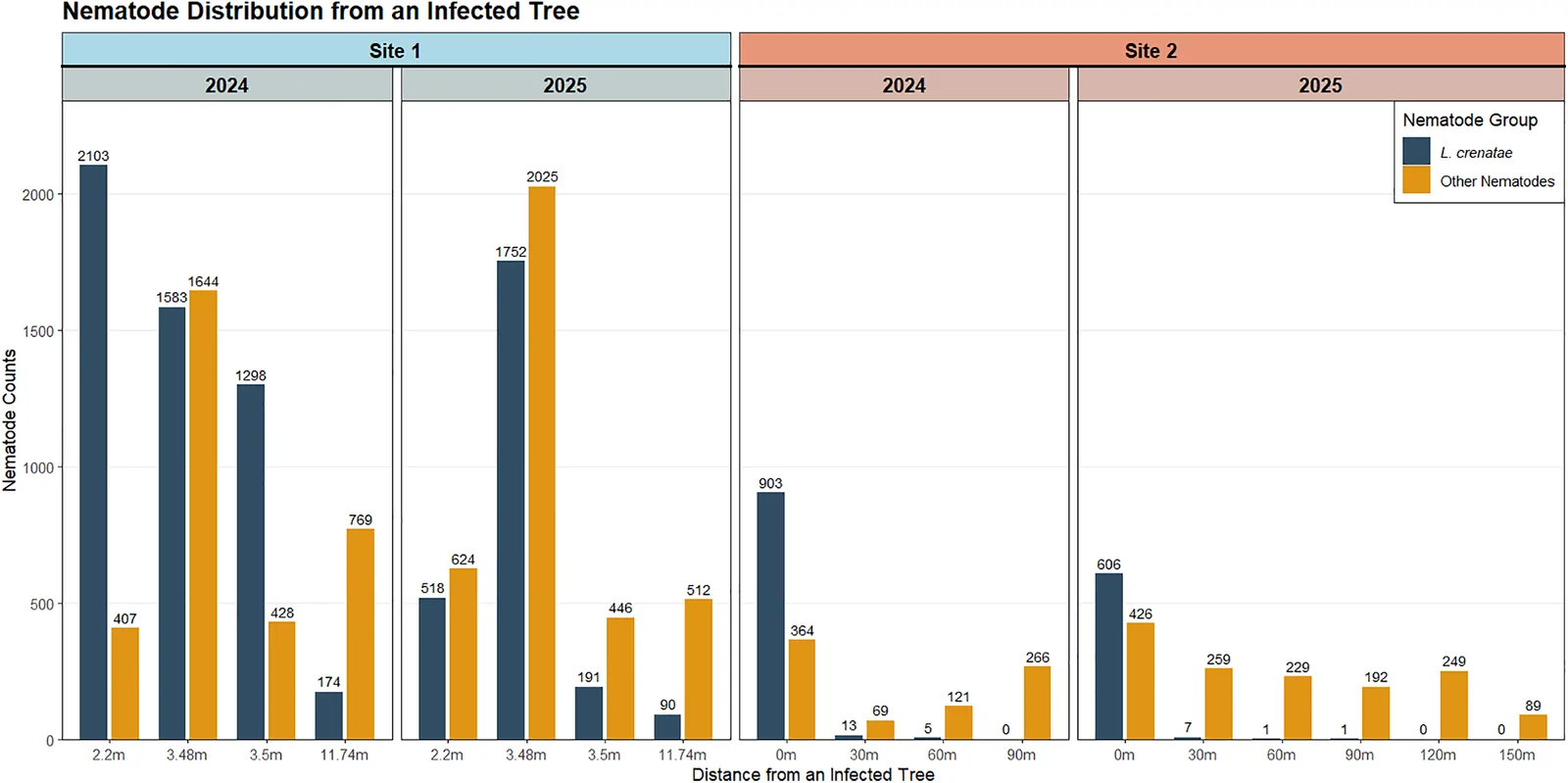
Counts of *Litylenchus crenatae* and other nematodes collected from the funnel traps placed at various distances away from an infected American beech tree. (Left) Total *L. crenatae* and other nematodes recovered at Site 1 from the 2024 and 2025 season. (Right) Total *L. crenatae* and other nematodes recovered at Site 2 from the 2024 and 2025 season.

Overall, Site 1 had significantly higher *L. crenatae* recovery rates compared to Site 2, due to the collection funnels’ closer proximity to the infected beech tree stand. On average, *L. crenatae* had a higher abundance in 2024 as compared to 2025, potentially due to the higher precipitation recorded that year. Funnel stands located 2.2 to 3.5 m away had similar rates of recovery for *L. crenatae*. However, there was a sharp decline of *L. crenatae* when the distance increased to 11.74 m away from an infected beech tree. Although *L. crenatae* recovery was still notable with an average of 132 *L. crenatae* captured at the funnels placed 11.74 m from the BLD stand, even though again this decline supports the distance-decay relationship between *L. crenatae* and an infected BLD stand.

#### Molecular confirmation of nematode identification.

Nematodes morphologically identified as *L. crenatae* were molecularly confirmed using the 28S rRNA gene. The amplification yielded fragments of 741 bp excluding primer binding sites. Consensus sequences were generated from the nematodes obtained from the funnels, water stream, bird feet wash, and UAV, were deposited in GenBank under accession numbers PZ575464, PZ575465, PZ575466, and PZ575467, respectively. The sequences generated for all specimens revealed 100% similarity to *L*. *crenatae* sequences obtained from specimens collected from other geographical areas in the US and Japan (GenBank accession numbers: OP689710.1, MK292137.1, and LC383725.1), therefore, validating our morphological analyses.

#### Influence of abiotic variables on *L. crenatae* dispersal.

Generalized linear mixed-effects models with a negative binomial distribution were used to evaluate the effects of environmental variables on *L. crenatae* dispersal at Site 1 and Site 2 study locations. Model diagnostics indicated an acceptable fit for both datasets. At Site 2, residual diagnostics detected no significant overdispersion (*p* = 0.792) or zero inflation (*p* = 0.584). Similarly, the Site 1 model showed no evidence of zero inflation (*p* = 0.496). Although a minor dispersion effect was detected at Site 1 (*p* = 0.024), residual diagnostics indicated acceptable model performance. Multicollinearity among predictors was low to moderate (VIF < 3.5), supporting inclusion of all environmental variables in the analyses.

*Litylenchus crenatae* dispersal was influenced by relative humidity, temperature, leaf wetness index, and precipitation (Table 1). Relative humidity was the strongest and most consistent predictor of *L. crenatae* recovery across both experimental sites (*p* < 0.001; Table 1). Temperature was negatively associated with nematode recovery (*p* < 0.001), indicating reduced *L. crenatae* abundance under warmer conditions. Leaf wetness index (LWI) positively affected *L. crenatae* recovery at both sites (*p* = 0.01 and *p* = 0.001 for Site 1 and Site 2, respectively), further supporting the importance of moisture-related conditions in facilitating nematode dispersal. Precipitation significantly influenced *L. crenatae* recovery at Site 1 (*p* = 0.01), whereas no significant effect was detected at Site 2 (*p* = 0.59). Wind-related variables were not significant and were excluded from the final models.

**Table 1:** Likelihood ratio test statistics (Chisq), degrees of freedom (Df), and associated P-values for the effects of precipitation, temperature, relative humidity, and leaf wetness index (LWI) on *Litylenchus crenatae* abundance at Site 1 and Site 2 field locations.

	Chisq		Df		Pr > Chisq	
Site	Site 1	Site 2	Site 1	Site 2	Site 1	Site 2
Precipitation	6.11	0.28	1	1	0.01*	0.59
Temperature	16.90	12.56	1	1	< 0.0001***	< 0.0001***
Relative humidity	21.57	17.89	1	1	< 0.0001***	< 0.0001***
Leaf wetness index (LWI)	6.24	9.92	1	1	0.01*	0.001**

Significance codes: p < 0.0001 ‘***’, p < 0.001 ‘**’, p < 0.01 ‘*’, p = 0.05 ‘.’

Complementary, a random forest model with variable importance was utilized to select the most significant factors influencing the overall spread and distribution of *L. crenatae*. The model identified the distance from the infected trees as the most important predictor of *L. crenatae* recovery, with traps closer to infected stands consistently capturing higher nematode numbers. Among the weather variables recorded, relative humidity had the highest score, with a mean decrease in accuracy (MDA) similar to that of distance (12.37% and 12.39%, respectively; Fig 4). Precipitation also showed high importance (MDA = 11.8%), indicating a strong association between rainfall events and *L. crenatae* recovery. Lastly, seasonality also influenced *L. crenatae* movement. Higher recoveries consistently occurred during spring and fall, corresponding to periods of bud presence and development, and leaf senescence. The Collection Date showed high MDA values for the random forest models, supporting the importance of seasonal patterns in *L. crenatae* dispersal.

**Fig 4:**
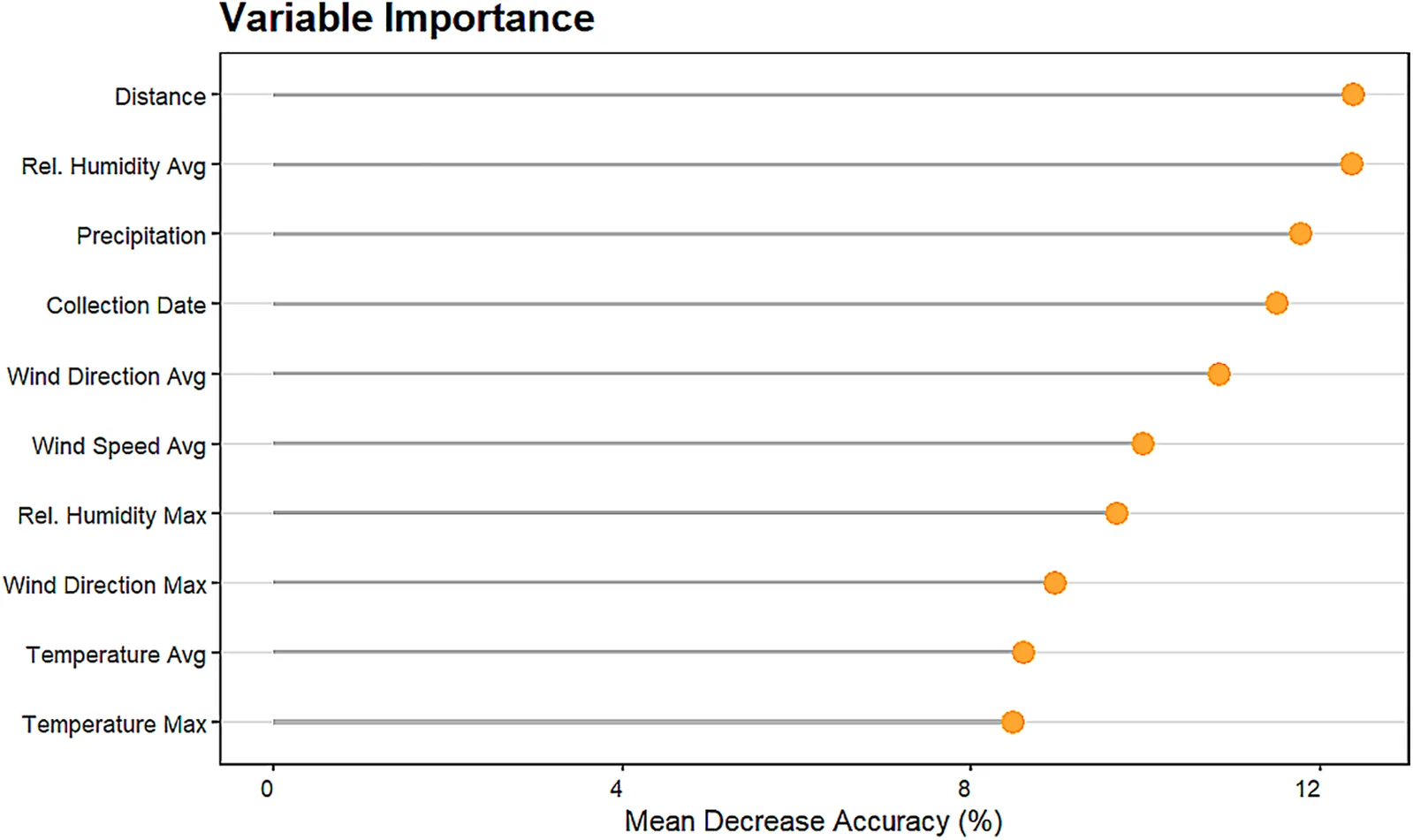
Random forest model predicting *Litylenchus crenatae* abundance based on local environmental factors, with variable importance rankings.

At both Site 1 and Site 2, peaks in *L. crenatae* recovery closely followed periods of elevated precipitation and high relative humidity (Figs 5 and 6). At Site 1 (Fig 5), the highest *L. crenatae* counts in 2024 occurred between September 24 and October 1, coinciding with rainfall events and relative humidity levels above 85% and funnel collections exceeding 1,900 *L. crenatae* specimens over a week. In 2025, recovery peaks again followed precipitation events, although *L. crenatae* counts were lower overall than in 2024. These observations are consistent with the statistical analyses, which identified relative humidity as one of the strongest predictors of *L. crenatae* counts (*p* < 0.001).

**Fig 5:**
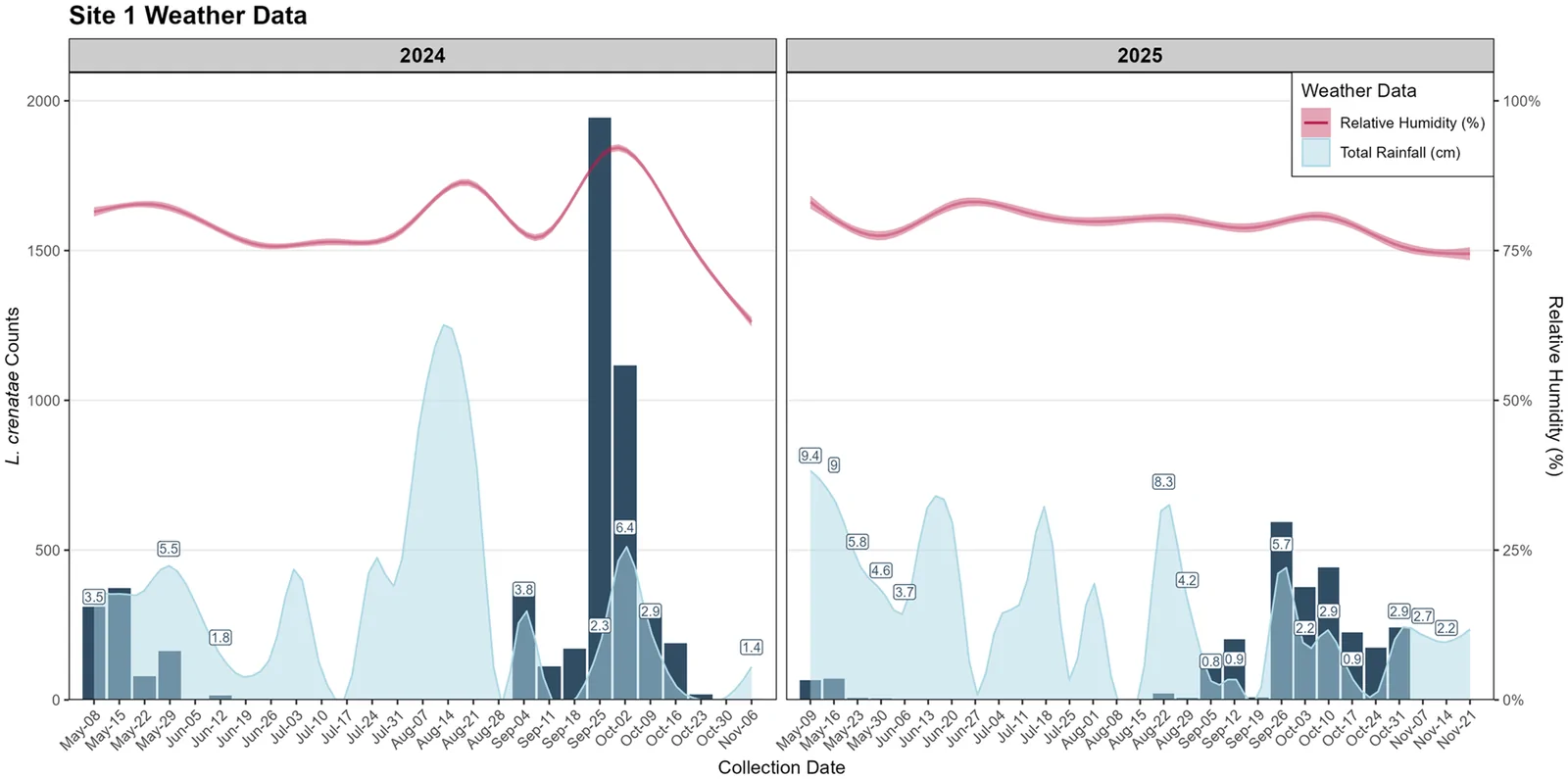
Local weather conditions and *Litylenchus crenatae* recovery at Site 1 in 2024 (Left) and 2025 (Right) during short-distance dispersal observations. Dark blue bars indicate the total number of *L. crenatae* collected at each date. The red line tracks the relative humidity (%) and the light blue bars track the total rainfall (cm) on each collection date.

**Fig 6:**
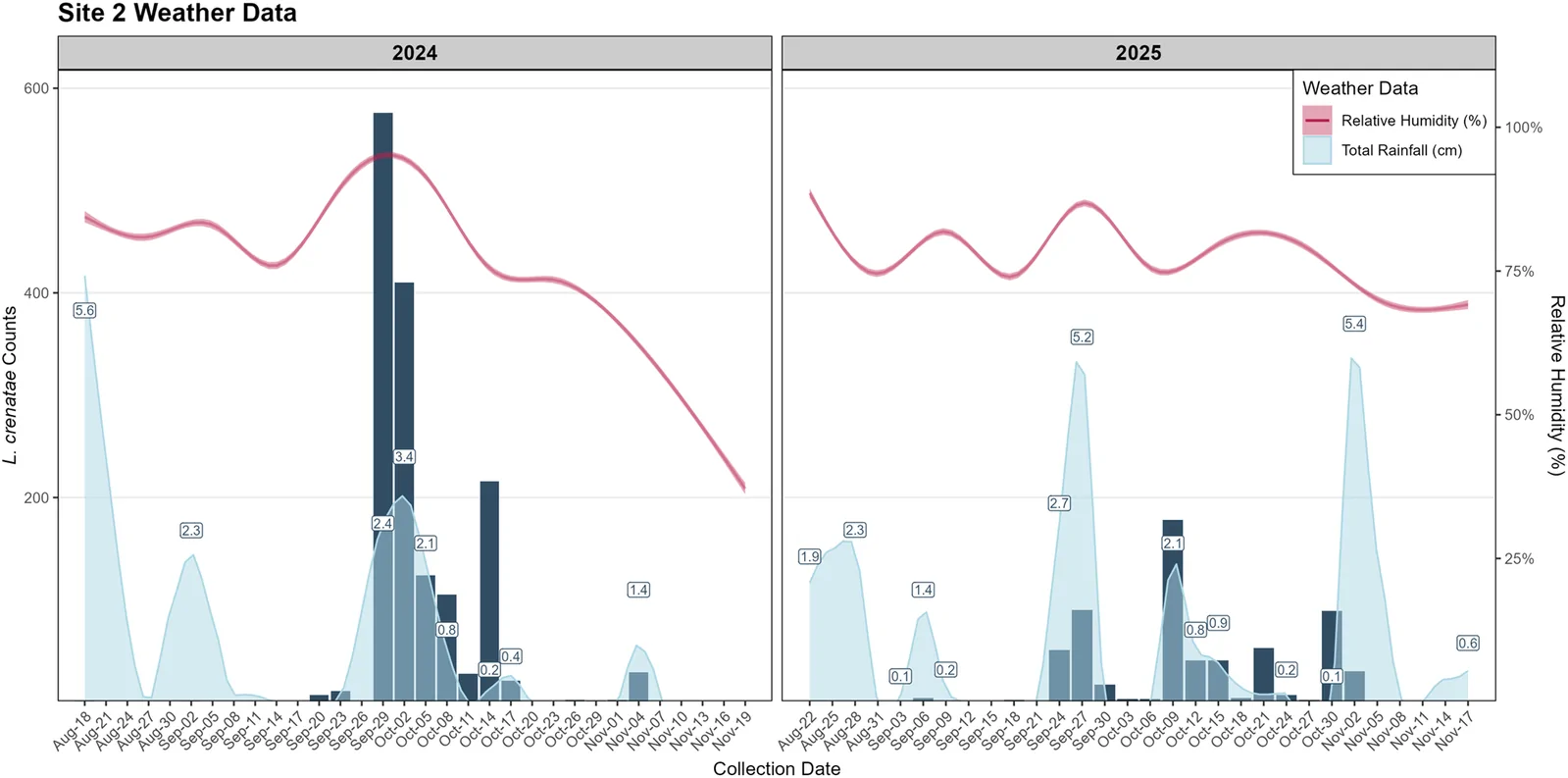
Weekly local weather patterns and *Litylenchus crenatae* recovery at Site 2 in 2024 (left) and 2025 (right). Dark blue bars indicate the total number of *L. crenatae* collected at each date. The red line tracks the relative humidity (%) and the light blue bars track the total rainfall (cm) on each collection date.

Similar trends were observed at Site 2 (Fig 6). The largest recovery peak occurred between September 29 and October 9, 2024, following multiple rainfall events. Relative humidity remained consistently high (>85%) during this period, supporting the strong positive relationship between moisture conditions and *L. crenatae* dispersal. Smaller recovery peaks were also observed after rainfall events in early November. In 2025, *L. crenatae* counts were lower, but increased recovery again coincided with precipitation events and elevated humidity.

#### Assessment of *L. crenatae* aerial dispersal using unmanned aerial vehicles.

*Litylenchus crenatae* were recovered in two of the three unmanned aerial vehicles (UAV) sampling events in the fall of 2025 (Table 2). The first sampling date, October 8, yielded the highest number of active *L. crenatae* recovered across all altitudes, with a total of 30 individuals collected. At 2 m altitude, 7 *L. crenatae* were recovered from two flights, whereas 23 *L. crenatae* were recovered at 15 m from the same number of flights. No nematodes were detected at 30 m, which was approximately 10 m above the tree canopy. During the second sampling event on October 30, lower numbers of *L. crenatae* were recovered, with 2 and 3 individuals collected at 2 m and 15 m, respectively, across three flights per altitude. Again, no *L. crenatae* were recovered at 30 m. The final sampling event, conducted on November 14, well past the end of the *L. crenatae* dispersal period shown in Figs 5 and 6, resulted in no *L. crenatae* or other nematodes recovered at any altitude. Across all sampling dates, *L. crenatae* were consistently recovered only at the lower altitudes (2 m and 15 m), whereas no individuals were detected at 30 m.

**Table 2:** Vertical dispersal of *L. crenatae* and other nematodes collected using UAV flights at 2, 15, and 30 m above ground level. Sampling was conducted 12 m from the trunk of a symptomatic American beech tree on three precipitation events (October 8, October 30, and November 14, 2025). The table summarizes the number of unmanned aerial vehicle (UAV) flights conducted at each altitude and the total numbers of *L. crenatae* and other nematodes recovered.

Flight Date	Altitude	Number of Flights	Total *L. crenatae*	Total other nematodes
10/08/2025	2 m	2	7	0
	15 m	2	23	4
	30 m	1	0	0
10/30/2025	2 m	3	2	1
	15 m	3	3	0
	30 m	3	0	2
11/14/2025	2 m	1	0	0
	15 m	1	0	0
	30 m	1	0	0

### Long distance dispersal

#### Assessment of *L. crenatae* dispersal via water streams.

Fig 7 illustrates the water stream-mediated dispersal of *L. crenatae* and other nematodes at a water stream near Site 1 from September to November 2025. Both *L. crenatae* and other nematode taxa, including free-living and plant-parasitic nematodes, were recovered in the sieves. Active *L. crenatae* were recovered primarily between September 19 to October 10, with peak recovery occurring on September 26, 2025, when 81 *L. crenatae* were collected. Notably, greater numbers of *L. crenatae* were recovered from 400 m downstream (55 *L. crenatae*) than directly below the infected stand (19 *L. crenatae*), indicating that water streams facilitate downstream dispersal. Other collected nematodes showed a similar pattern, with the highest recoveries throughout September. By late November, nematode recoveries were minimal, and only inactive *L. crenatae* were detected. These results suggest that creeks and streams may contribute to long-distance dispersal of *L. crenatae*.

**Fig 7:**
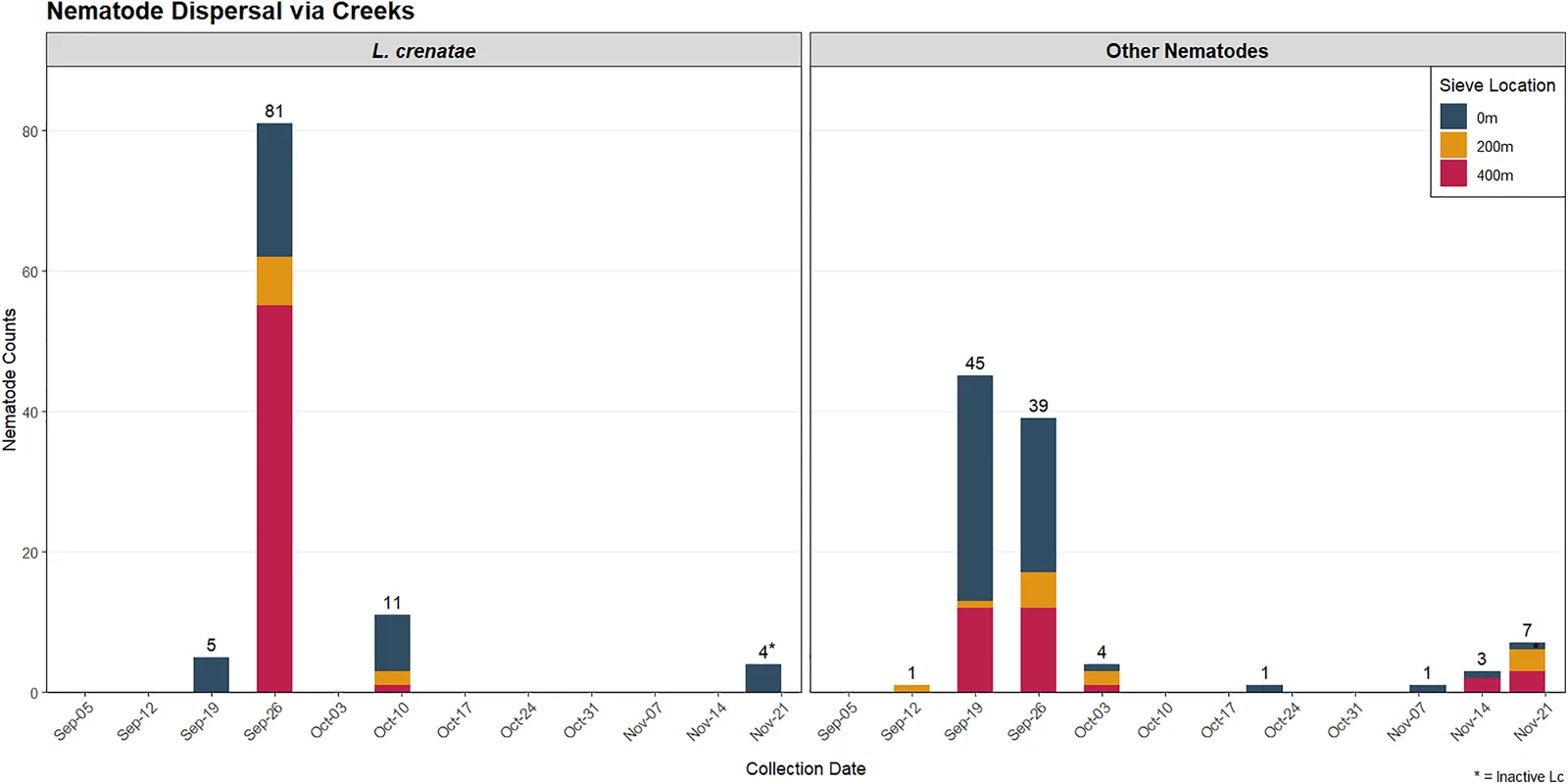
Water stream-associated dispersal of *Litylenchus crenatae* and other nematodes throughout the Fall 2025 season near Site 1 tracked across 3 downstream locations.

#### Assessment of bird-mediated nematode dispersal.

A total of 29, 47, and 62 individual birds were sampled, and their feet were washed during sampling events conducted on September 5, September 23, and October 15, 2025, respectively. Nematodes were recovered from six migratory bird species, including *L. crenatae* and other nematodes (Table 3).

**Table 3:** Summary of birds carrying nematodes collected on September 23, 2025.

Scientific name	Common name	Total number of individuals sampled	Number of nematode-carrying birds	Total number of *L. crenatae* and other nematodes recovered
*Setophaga citrina*	Hooded Warbler	4	2	Bird 1: 1 *L. crenatae*
				Bird 2: 3 *L. crenatae*
*Setophaga magnolia*	Magnolia Warbler	11	5	Bird 1: 1 *L. crenatae*
				Bird 2: 1 *L. crenatae*
				Bird 3: 1 *L. crenatae*
				Bird 4: 1 Other nematode
				Bird 5: 1 Other nematode
*Parkesia noveboracensis*	Northern Waterthrush	3	1	Bird 1: 1 *L. crenatae*
*Melospiza lincolnii*	Lincoln’s Sparrow	3	1	Bird 1: 2 *L. crenatae*
*Passerina cyanea*	Indigo Bunting	1	1	Bird 1: 1 Other nematode
*Leiothlypis peregrina*	Tennessee Warbler	2	1	Bird 1: 1 Other nematode
**Total**				10 *L. crenatae*, 4 Other nematodes

Nematodes were detected only during the September 23 sampling event, when 10 *L. crenatae* individuals and four other nematodes were recovered from 21.6% of the birds sampled on that date. The Magnolia Warbler (*Setophaga magnolia*) was the species most frequently harboring nematodes, whereas the highest number of *L. crenatae* recovered from a single bird was three individuals on a Hooded Warbler (*Setophaga citrina*). Additional *L. crenatae*-positive species included the Northern Waterthrush (*Parkesia noveboracensis*) and Lincoln’s Sparrow (*Melospiza lincolnii*). Other nematodes, including one *Laimaphelenchus* sp., were also recovered from several migratory bird species. All nematodes classified as other nematodes were alive at the time of quantification, whereas all *L. crenatae* individuals were dead during quantification. No nematodes were recovered during the September 5^th^ and October 15^th^ sampling events, which was characterized by the lowest number of birds recovered and high morning humidity in the absence of rainfall, respectively.

#### Greenhouse simulation of bird-mediated beech infection.

Greenhouse trials simulating bird-mediated dispersal demonstrated that *L. crenatae* can be transferred to healthy beech trees through contaminated bird feet. In the first experiment, detached quail feet carrying an average of 15 *L. crenatae* per contact were randomly applied 10 times across branches of 15 beech trees. Five trees (33% of the trees) were later found positive for *L. crenatae* in buds, although most infected buds contained only a single inactive nematode.

In a second experiment, quail feet were applied directly near apical buds three times per tree, increasing inoculation success. Seven of 15 trees (46.7%) contained *L. crenatae*-positive buds, including one tree with both inactive and active adult *L. crenatae* individuals. No juveniles or eggs were detected in any buds.

Following bud break in spring, characteristic BLD leaf banding developed in all five trees infected during the random inoculation treatment, and in 11 of the15 trees (73.3%) inoculated near apical buds (S3 Fig). These results suggest that direct deposition of *L. crenatae* near buds substantially increases infection success and supports the potential role of birds in BLD dispersal.

#### Assessment of *L. crenatae* desiccation tolerance.

Desiccation time significantly affected *L. crenatae* activity following rehydration (GLMM, binomial distribution, *p* < 0.001). The proportion of active nematodes declined progressively with increasing drying duration (Fig 8). During the early desiccation intervals, *L. crenatae* movement remained relatively high, with mean activity levels of 81.8% after 1 h and 64.6% after 2 h of drying. Activity continued to decline at 5 h, where approximately 49.3% of recovered nematodes remained active after rehydration. A marked reduction in nematode activity occurred 24 h after desiccation, with mean activity decreasing to 11.1%. Lower but detectable levels of *L. crenatae* activity persisted throughout the remaining sampling periods, averaging 8.7% at 48 h, 12.0% at 72 h, 2.7% at 120 h, and 2.3% after 240 h (10 days) of desiccation. Pairwise comparisons indicated that nematode activity at 24 h and longer desiccation periods was significantly lower than activity observed during the first 5 h of drying (Tukey-adjusted, *p* < 0.001). Despite the substantial decline in activity over time, active nematodes were recovered at all sampling intervals, demonstrating that *L. crenatae* can tolerate extended desiccation periods and potentially survive environmental conditions conducive to long-distance dispersal.

**Fig 8:**
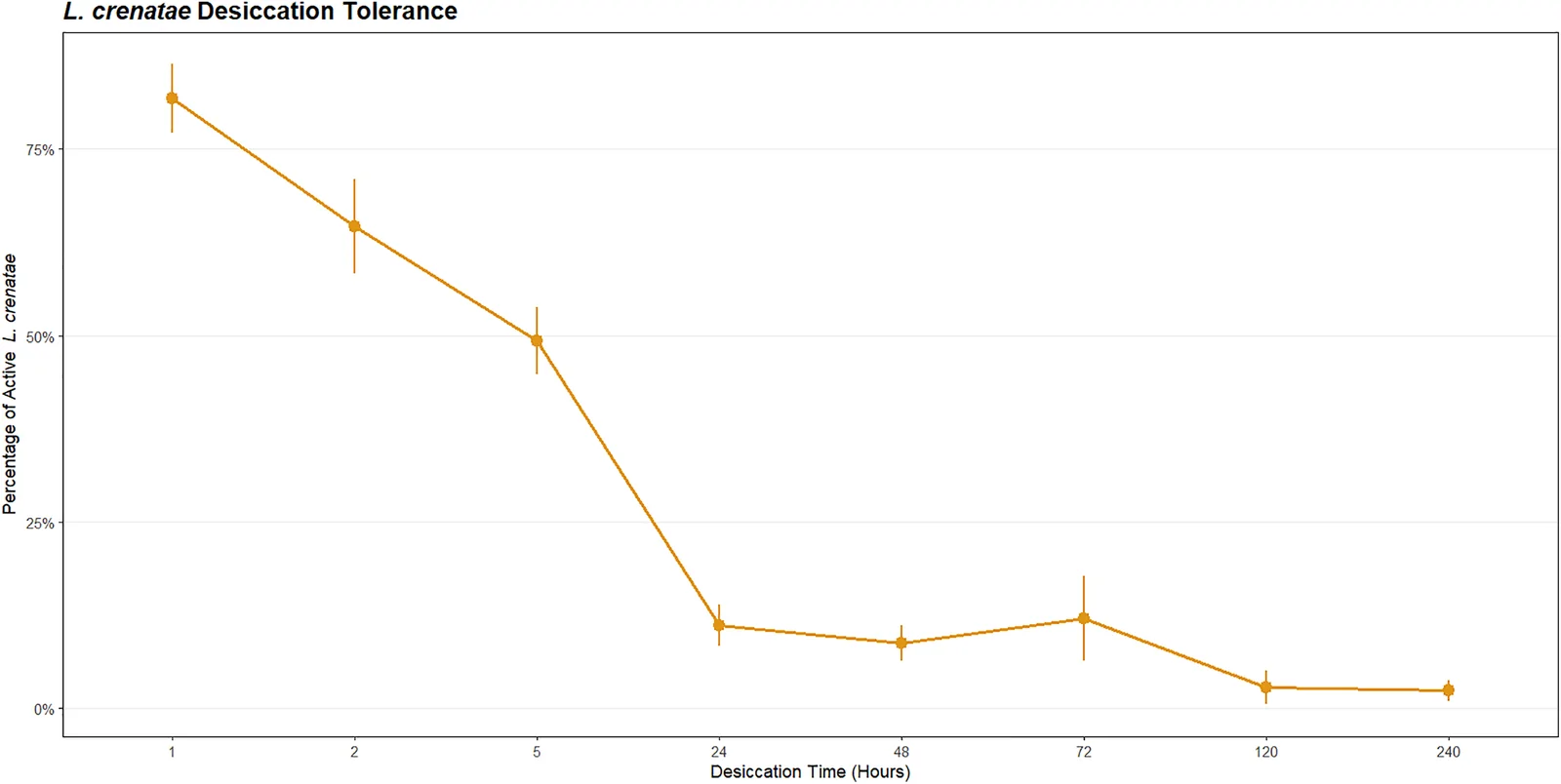
Desiccation tolerance of *Litylenchus crenatae* following rehydration. Bars indicate standard errors from three experiments.

## Discussion and conclusions

In forest ecosystems, the spatial movement of foliar plant parasitic nematodes remains poorly understood despite its major ecological implications. BLD is rapidly spread through Eastern hardwood forest systems, sweeping through the Mid-Atlantic region [2]; therefore, understanding the mechanisms behind BLD’s dispersal is ecologically critical [6]. *L. crenatae* infestation does more than just damage individual trees; it significantly alters the foliar microbiome, disrupts carbon and nutrient cycling, and forest succession across affected landscapes [23,24]. This study demonstrates that the spread of *L. crenatae* is a dynamic and multi-scale process strongly influenced by host phenology, environmental conditions, and multiple passive dispersal pathways.

Through multi-year monitoring across forest sites, we identified consistent seasonal patterns of *L. crenatae* movement, characterized by pronounced dispersal peaks during spring bud break and fall senescence, with minimal movement observed during summer. These findings support previous observations that *L. crenatae* overwinters within dormant buds before migrating into newly emerging foliage in spring [11]. In contrast, dispersal activity declined during summer, likely reflecting completion of leaf colonization and reduced suitability of mature leaf tissues for nematode invasion. The consistency of emergence periods across years suggests that *L. crenatae* movement is closely synchronized with host phenology and may be predictable based on seasonal bud development.

Fall dispersal was substantially greater than spring dispersal, with peak *L. crenatae* recovery occurring during September and October. During this period, senescing leaves likely serve as major inoculum sources through leaf abscission, rain-mediated wash-off, and wind-driven movement. Although American beech is typically a marcescent species, BLD infection can accelerate leaf senescence, and induce premature leaf drop [25,26]. This altered phenology increases the volume of infected leaf litter inoculum on the forest floor during the autumnal months. Additionally, this fall dispersal peak coincides with overwintering bud formation, potentially promoting nematode migration into newly developing buds prior to tree dormancy [11]. Together, these observations suggest that seasonal canopy turnover plays a major role in amplifying the landscape-level spread of BLD.

Distance from infected beech trees was the strongest predictor of *L. crenatae* abundance across both generalized linear mixed models and random forest analyses, supporting a classic distance-decay dispersal pattern commonly observed in seeds and plant pathogen dispersal systems [26,27]. Funnel stands located near symptomatic trees consistently recovered greater numbers of nematodes, whereas recoveries declined substantially with increasing distance. Nevertheless, active *L. crenatae* individuals were recovered up to 90 m from infected trees, demonstrating that the nematode can disperse well beyond immediate host proximity. Although long-distance recoveries occurred at relatively low densities, even infrequent dispersal events may contribute substantially to disease expansion over time. This is particularly important because the transport of only small numbers of nematodes may be sufficient to initiate local population establishment and subsequent disease development, although the minimum infective dose remains unknown. Similar low-density, long-distance dispersal events are recognized as critical drivers of invasion dynamics in emerging forest diseases [28,29].

Environmental conditions, including relative humidity and precipitation, also strongly influenced local dispersal dynamics. These findings support the hypothesis that moisture availability is critical for above-ground nematode movement and survival, as thin water films are required for nematode mobility and protection against desiccation [30,31]. Similar associations between rainfall, wind events, and short-distance *L. crenatae* dispersal were previously reported by [12].

In our study, several long-distance *L. crenatae* recovery events coincided with periods of elevated rainfall and moderate wind speeds, suggesting that moisture enhances nematode survival during transport, while wind facilitates directional movement through the canopy and understory. Interestingly, wind was a significant predictor of *L. crenatae* recovery at the short-distance from Site 1, but not at the longer-distance as in Site 2, indicating that the influence of wind on dispersal may vary depending on spatial scale and local forest structure.

Unlike root-feeding plant-parasitic nematodes, whose movement is largely constrained to the soil profile and often influenced by human-mediated transport, foliar nematodes occupy a far more complex three-dimensional environment. Species such as *Aphelenchoides besseyi*, *A. fragariae*, and *Bursaphelenchus xylophilus* rely on water films, rain splashes, wind currents, or vector-mediated movement to disperse among hosts and canopy layers [31]. In forest ecosystems, canopy architecture and vertical stratification influence microclimatic conditions that affect nematode survival and dispersal potential.

In the case of BLD, *L. crenatae* movement occurs not only horizontally between trees, but also vertically throughout forest canopies, understory vegetation, and leaf litter layers. Our UAV-assisted canopy sampling revealed that *L. crenatae* dispersal was concentrated within the lower to mid-canopy range, with the highest recoveries occurring near 15 m, and no detections observed above canopy level at 30 m. These findings suggest that vertical movement up above tree canopies might be constrained by decreasing humidity with altitude and local wind patterns reducing the chance of *L. crenatae* to travel up above the emergent layer, further supported by the relative absence of other nematode species at this altitude. However, since the UAV study was conducted during relatively few rain events, additional multi-year sampling under sustained rainfall or high humidity conditions will be necessary to fully characterize vertical dispersal dynamics within mature forest canopies.

Recovery of *L. crenatae* from water stream systems demonstrated that hydrological networks can transport nematodes over considerable distances, as active *L. crenatae* were recovered up to 400 m downstream from infected stands. These findings highlight creeks and waterways as potentially important landscape-level dispersal corridors for BLD. Moving water has increasingly been recognized as an efficient, yet often underestimated, dispersal mechanism for plant-parasitic nematodes [6]. Heavy rainfall and runoff events can help to mobilize nematodes from leaf litter, soil surfaces, and low-hanging foliage into watershed systems, allowing passive transport far beyond their active migration capacity [31,32].

In our study, *L. crenatae* recovery from stream systems occurred primarily during September and October, coinciding with peak nematode activity and the onset of leaf senescence. This seasonal association suggests that although water may facilitate highly effective long-distance transport, successful dispersal likely depends on favorable environmental conditions for nematodes migration and the availability of infected senescing foliage as inoculum sources. Because American beech commonly occurs in mesophytic forests with abundant soil moisture and stream networks [23], hydrological dispersal may contribute substantially to local and regional disease spread [33].

Our findings also support the hypothesis that biotic vectors may contribute to long-distance movement of *L. crenatae* across fragmented forest landscapes. Vector-mediated dispersal is well documented in other forest nematode systems, most notably in the pine wood nematode, *Bursaphelenchus xylophilus*, which relies on *Monochamus* beetles for transmission between hosts [34]. For BLD, [4] first proposed that mites and birds could act as phoretic vectors of *L. crenatae*. Subsequent studies recovered *L. crenatae* from predatory mites and from tussock moth frass and spider webs [12], further supporting the possibility of vector-mediated dispersal.

In the present study, the recovery of *L. crenatae* and other live free-living nematodes from migratory bird wash samples, together with successful greenhouse inoculation trials simulating bird-mediated deposition, support the potential role of birds in BLD dispersal. Although recovered *L. crenatae* individuals from bird feet were inactive at the time of observation, the presence of live free-living nematodes demonstrates that nematodes can persist externally on bird surfaces during transport. Bird species associated with *L. crenatae*, including the Magnolia Warbler, Hooded Warbler, and Lincoln’s Sparrow, possess migratory and breeding ranges spanning much of eastern North America and the current BLD infection range, potentially providing opportunities for landscape-scale movement among disconnected forest patches.

While relatively few studies have examined the external transport of nematodes by birds, migratory birds are known to harbor diverse nematode communities and contribute to parasite dispersal across broad geographic regions [35]. Birds associated with aquatic habitats, such as Lincoln Sparrows which breed in wetter habitats, may be particularly important dispersal agents because of their increased exposure to moist environments favorable for nematode survival [35,36].

Finally, desiccation assays demonstrated that *L. crenatae* possesses substantial tolerance to drying, with viable nematodes recovered after up to 10 days of desiccation under controlled humidity conditions. This ability to survive prolonged periods of low moisture through anhydrobiosis likely represents a key ecological adaptation, facilitating survival on exposed surfaces such as leaf litter, tree bark, bird feet, arthropods, or canopy foliage where nematodes are subjected to fluctuating temperature and humidity conditions prior to reaching a new host. Similar adaptations have been reported in other anguinid nematodes, with physiological and metabolic changes allowing survival under extended dehydration stress [37]. The capacity of *L. crenatae* to remain viable under dry conditions likely increases the probability of successful long-distance dispersal and establishment following transport by wind, water, or biotic vectors.

These findings demonstrate that BLD spread is driven by a complex, multi-scale dispersal network rather than a single mode of transmission. More specifically, BLD transmission is governed by the interaction of seasonal host phenology, spatial limitations, environmental conditions, and multiple abiotic and biotic dispersal mechanisms, helping explain the rapid expansion of this disease across eastern North American forests. While local spread is strongly governed by a classic distance-decay relationship and facilitated by wind and moisture; *L. crenatae* is also capable of significant long-distance dispersal. This extended reach is enabled by a combination of hydrological transport through forest streams networks, potential phoretic by migratory birds, and the nematode’s anhydrobiosis capability, allowing it to survive extended periods of environmental stress during migration. Therefore, *L. crenatae* can occupy a dynamic three-dimensional environment, moving both horizontally across fragmented landscapes and vertically within the lower and mid-forest canopy.

When considering that *L. crenatae* dispersal is facilitated by multiple intersecting abiotic and biotic pathways, traditional management approaches focused solely on localized containment will likely be insufficient. Effective strategies for tracking and mitigating BLD spread must account for these diverse, landscape-level dispersal mechanisms particularly via watershed corridors, seasonal weather events, and avian vectors to better predict and manage the trajectory of this devastating forest epidemic. Understanding how these pathways interact and contribute to *L. crenatae* nematode movement will be essential for predicting future forest-pathogen dynamics, identifying high risk areas for BLD monitoring, and the development of effective management strategies for current and future forest systems.

## Supporting information

S1 Fig Assessment of *L. crenatae* aerial dispersal using unmanned aerial vehicles setup. (A) Unmanned Aerial Vehicle experimental set-up; (B) Unmanned Aerial Vehicle flights at 5 m altitude. The arrow in panel B indicates the position of the collection chambers.
(TIFF)

S2 Fig Sieve box set used to assess *Litylenchus crenatae* dispersal through stream water. (TIFF)

S3 Fig American beech leaves infected in the greenhouse using the quail foot assay exhibiting characteristic Beech Leaf Disease banding, along with crinkling and deformation. (TIFF)
